# The dysregulation of tRNAs and tRNA derivatives in cancer

**DOI:** 10.1186/s13046-018-0745-z

**Published:** 2018-05-09

**Authors:** Shi-qiong Huang, Bao Sun, Zong-ping Xiong, Yan Shu, Hong-hao Zhou, Wei Zhang, Jing Xiong, Qing Li

**Affiliations:** 1 Department of Clinical Pharmacology, Institute of Clinical Pharmacology, Hunan Key Laboratory of Pharmacogenetics, Xiangya Hospital, Central South University, Changsha, Hunan 410078 People’s Republic of China; 2 Institute of Clinical Pharmacology, Hunan Key Laboratory of Pharmacogenetics, Central South University, Changsha, 410078 People’s Republic of China; 3 Department of Pharmaceutical Sciences, School of Pharmacy, University of Maryland at Baltimore, Baltimore, MD USA; 4 Department of gynaecology and obstetrics, The Second Xiangya Hospital of Central South University, Central South University, Changsha, 410078 People’s Republic of China

**Keywords:** tRNA, tRNA derivatives, Breast cancer, Lung cancer, Melanoma

## Abstract

Transfer RNAs (tRNAs), traditionally considered to participate in protein translation, were interspersed in the entire genome. Recent studies suggested that dysregulation was observed in not only tRNAs, but also tRNA derivatives generated by the specific cleavage of pre- and mature tRNAs in the progression of cancer. Accumulating evidence had identified that certain tRNAs and tRNA derivatives were involved in proliferation, metastasis and invasiveness of cancer cell, as well as tumor growth and angiogenesis in several malignant human tumors. This paper reviews the importance of the dysregulation of tRNAs and tRNA derivatives during the development of cancer, such as breast cancer, lung cancer, and melanoma, aiming at a better understanding of the tumorigenesis and providing new ideas for the treatment of these cancers.

## Background

Not participating in protein-coding, small non-coding RNA (sncRNA), including transfer ribonucleic acids (tRNA), ribosomal RNA (rRNA), small nucleolar RNA (snoRNA) and small nuclear RNA (snRNA), plays a widespread and important role both inside and outside the cell and is involved in cell proliferation, differentiation, apoptosis, and cellular metabolism [1]. With the development of high-throughput sequencing technology, new types of sncRNAs are obtained from other cellular RNA species through specific and regulated RNA processing or cleavage [2–4]. For instance, the cleaved products of mature snoRNA were mainly derived from a single gene: the C/D box type snoRNA gene methylation guide foe U6 snoRNA residue 77 (mgU6–77) [5, 6]. Also, novel sncRNAs were identified by cleavage of anti-codon loops, TψC loops, D loops, and other positions of tRNAs [4].

Previously, it was a generally accepted fact that tRNA was a housekeeping product with little regulatory function and it was rarely known by researchers the additional function of tRNAs apart from their canonical function as adapters in protein synthesis [7]. However, there are growing evidence in recent years that tRNAs and their derivatives are dysregulated in cancer and involved in the pathogenic process of cancer, for which they have recently gained significant attention [8, 9]. Furthermore, mutation of the tRNA itself and the involvement of the supplementary protein produced by the tRNA biogenesis and modification is also associated with cancer [10–12]. In this review, current understanding of the dysregulation of tRNAs and tRNA derivatives in tumor pathogenesis was summarized in terms of main cancer types such as breast cancer, lung cancer, and melanoma.

## Biogenesis and structure of tRNAs and tRNA derivatives

### Biogenesis and structure of tRNAs

tRNAs, with fundamental function of carrying and transporting amino acids, are a class of small noncoding ribonucleic acid folded into a “clover” secondary structure and L-shaped three-stage structure composed of 70–90 nucleotides, accounting for approximately 4–10% of all cellular RNAs [13]. As an elementary composition of the translation procedure, they convey the amino acid to the ribosome and convert the significance of the nucleotide sequence to the corresponding polypeptide chain in a manner of the interaction of codon (mRNA)-anticodon (tRNA) [14, 15]. Initial transcription product of RNA polymerase III (Pol III) is a typical precursor of tRNA that obligatorily experiences a succession of intricate biological processes to be converted to mature tRNA [16, 17], including removal of the 5′ leader by RNase P, clipping of the 3′ trailer by endonucleases and exonucleases such as RNase E, RNase PH and RNase T, addition of CCA at the 3' end via CCA-adding enzyme, shearing of introns and multiple modifications of tRNA base [18, 19]. It is important that accurate processing of pre-tRNA is essential to its successful release from the nucleus to play part in the translation of the protein. However, not all tRNAs are used for protein translation and a small percentage of them play the role of signaling molecules in response to environmental stress [20]. Under oxidative stress, tRNAs were cleaved into small molecules RNAs that repressed translation initiation [21]. Equally, oxidative stress-induced deactivation of the 3' CCA tail also shut down global translation [22].

Previously, the changes in mRNA levels in cancer cells effectively explained why cancer cells proliferate, metastasize and avoid death in their own ways [23, 24]. Researchers currently found that alteration in transcriptional level did not necessarily mean the change of protein level [25, 26], and to some extent tRNA played an irreplaceable role in the translation of proteins. The abundance, modification, and mutation of tRNA are all closely related to the protein expression. It is unexpected that the synthesis of tRNA is controlled by all kinds of oncogenes and tumor suppressors—Ras [27] and c-myc [28] promote the transcription of RNA Pol III, whereas Rb [29] and p53 [30] inhibit RNA Pol III transcription, causing the serious dysregulation of tRNA level in a wide range of cancers. In addition, Gingold et al. suggested that there were two representative cellular states in a multicellular animal, namely cell proliferation and cell differentiation corresponded to two distinctly different active tRNA pools referred as proliferative tRNA pools (pro-tRNAs) and differentiated tRNA pools (dif-tRNAs) respectively [31]. Further analysis revealed that the codons abundant in the cancer cells and inducing differentiation condition of genes were corresponded to the induced pro-tRNAs and dif-tRNAs pools, respectively. The cohort of these two genes was due to their histological modification on the chromatin [31, 32]. Moreover, several studies had shown that tRNA-modifying enzymes increased modifications to specific tRNAs in several cancers, which altered the codon preference of the tRNA that in turn led to an increase in the protein expression levels of those mRNAs found to be wealthy with a particular subset of the new “preferred” codons [33, 34]. Gerber et al. provided the evidence that the activity of adenosine deaminase (ADATs; A-to-I transformation) expanded the wobbling capacities of the tRNA base at position 34, allowing it to pair with three different codons—A, U, C [35]. Mutations in mitochondrial tRNA caused mitochondrial dysfunction also involved in tumorigenesis [36]. Finally, the binding of tRNA to cytochrome c suppressed the action of cytochrome c and apoptosis protease catalysts, thereby inhibiting apoptosis and the activity of the enzyme [37].

### Biogenesis and structure of tRNA derivatives

Increasing evidence argues that tRNAs and tRNA derivatives are not only imperative ingredients of translation mechanism, but also significant signaling molecules in response to stress [38]. Furthermore, the earlier researchers reported that tRNA breakdown products existing in tumor tissue were often more frequent during stress [39], and it was noteworthy that they were observed in the sera and urine of cancerous person with expression level roughly related to cancer burden [40].

**Fig. 1:**
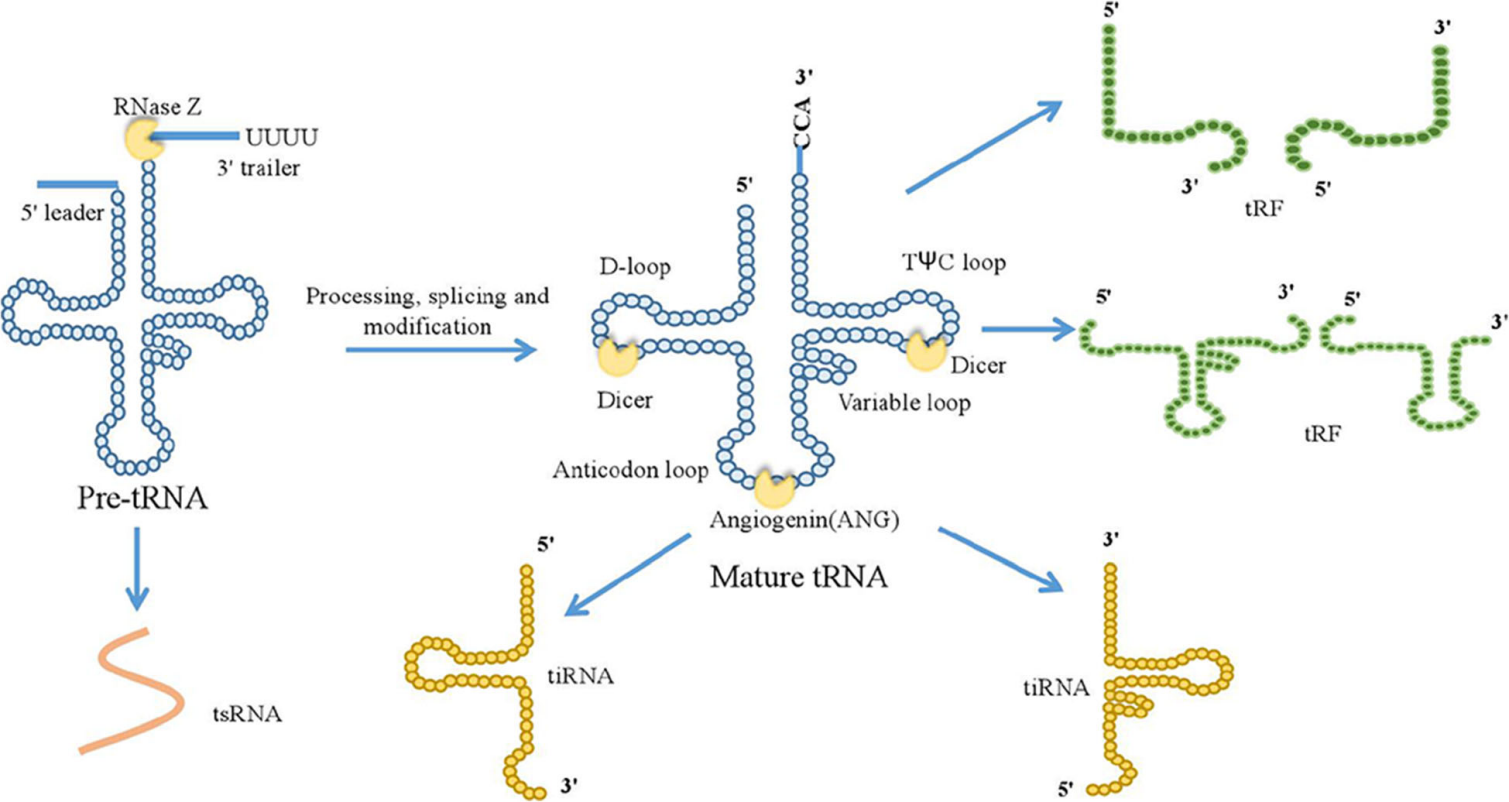
Different types of tRNA derivatives came from the cleavage of pre-tRNAs and mature tRNAs. tsRNAs were generated in the nucleus as a consequence of the pre-tRNA 3′ end cleavage. tiRNAs were generated as a consequence of angiogenin cleaving the anti-codon loop of the mature tRNA. tRFs were formed by Dicer at D-loop, T-loop and other positions of the nucleic acid ribozyme through cleavage of the mature tRNA

A wealth of intriguing studies discovered that tRNA derivatives, including tRNA-derived stress-induced RNAs (tiRNAs) [41, 42], tRNA-derived fragments (tRFs) [43], and tRNA-derived small RNAs (tsRNAs) [44], were generated by cleavage of the pre-tRNAs or mature tRNAs under various environmental stresses [45, 46] (Fig. 1).

Not purely tRNA degradation debris, tRNA derivatives had a vital role in cancer, RNA silencing and micro-environment monitoring [47]. tiRNAs, including 5' tiRNAs and 3' tiRNAs, were generated as a consequence of angiogenin cleaving the anti-codon loop of the mature tRNA [21]. 5' tiRNAs was interacted with tRNase Z^L^ (tRNA endonuclease) binding to the enzyme to cleave the target gene complementary to the 5′ tiRNAs sequence where 5' tiRNAs acted as a small guide RNA, thereby down-regulating the expression of the target gene [48, 49]. Several studies, furthermore, have reported that tiRNAs inhibit protein synthesis and induce the formation of stress particles [21, 50]. tRFs are relatively smaller RNAs (17-26 nt) formed by Dicer or Rnase Z at other positions of the nucleic acid ribozyme through cleavage of the mature tRNA [51]. tRFs can form a complex with Argonaute (Ago) protein, and play a similar role with miRNA by silencing a series of target gene expression [52, 53]. The high expression of the tRF in an extensive range of cancer cell lines is strongly relevant to cell proliferation [51]. tsRNAs come into being through cleaving the pre-tRNA 3'end [44]. Their conspicuous dysregulation, which may exert oncogenic or tumor-suppressor functions in cancer, have been discovered in a variety of malignant tumor onsets and progression [44, 54].

## tRNAs and tRNA derivatives in breast cancer

### The overexpression of tRNAs in breast cancer

Breast cancer is a fairly common malignant tumor that occurs in the glandular epithelial tissue, whose incidence has been a continuous upward trend since the late 1970s and ranks first in female malignancies [55]. Recently, the research on tRNA and breast cancer is increasing [8, 56, 57]. Mahlab et al. observed that the absolute levels of tRNA genes in breast cancer cells were significantly increased compared with healthy cells [56]. Nuclear- and mitochondrial-encoded tRNAs, also upregulated in breast tissues, were greatly enhanced in cancer cell lines based on genome-wide studies of tRNA expression [8]. tRNA profiling revealed that tRNA expression levels in breast cancer lines were different from those of non-tumorigenic cells [57].

What’s the reason for the increase in tRNA levels in cancer cells/tissues? Several pieces of evidence showed that transcription of tRNA by RNA pol III was affected by proto-oncogenes and tumor suppressor genes, which mainly impacted the subunit Brf1 of TFIII B factor in pol III, thus promoting or suppressing its function [58–60]. In estrogen receptor (ER)-positive breast cancer, the interaction of estrogen receptor alpha (ERα) with Brf1 mainly regulated the transcription of the Pol III genes—particularly tRNA^Leu^ and 5S rRNA [61]. In addition, inhibition of ERα not only reduced the expression of Brf1 and Pol III gene but also decreased the formation rate of breast cancer cell colony [61]. The researchers discovered a significantly positive correlation between the expression of telomerase reverse transcriptase (TERT) and pretranscripts of tRNA-Leu and tRNA-Tyr only in triple-negative breast cancer with highly aggressive ability by correlation analysis [62, 63]. Moreover, loss of TERT was related to reduced polyomavirus middle T oncogene-induced (PyMT-induced) mammary tumorigenesis and expression of tRNA such as pre-tRNA-Tyr [62, 64]. Recent studies suggested that all tRNA^Leu^ isoacceptors were more highly expressed only in human epidermal growth factor receptor-2 (Her2) -positive breast subtypes, but not in other subtypes, which greatly promoted the proliferation of cancer cells and their resistance to death via interaction of tRNA^Leu^_CAG_ with ErbB3-binding protein 1 (EBP1), subsequently enhancing the activation of ErbB2/ErbB3 pathway and the RSK1/MSK2 signaling pathway [65].

Furthermore, increased tRNAi^Met^ in human mammary epithelial cells could enhance the capacity of cell proliferative and metabolism [66]. But there was a poor correlation between the tRNA levels induced by tRNAi^Met^ overexpression in mammary epithelial cells and that levels in breast cancer cells, suggesting that the change of tRNA abundance and species in cancer cells had little to do with the overexpression of tRNAi^Met^ [66]. Furthermore, Clarke et al. detected that increased tRNAi^Met^ in carcinoma-associated fibroblasts could promote tumor growth and angiogenesis [67]. They measured tRNA levels in immortalized human breast cancer-associated fibroblasts (iCAFs) and normal fibroblasts and observed an increase in the expression of tRNAi^Met^ and tRNA^Ile^ in iCAFs [67, 68].

It was surprising that overexpression of tRNA also affected the ability of invasion and metastasis in cancer cells [57]. Using means of a new tRNA profiling method, investigators revealed that specific tRNAs, tRNA^Arg^_CCG_ and tRNA^Glu^_UUC_, were upregulated in highly metastatic breast cancer cells compared to poorly metastatic counterpart due to their enhanced stability and translation of the codon-rich transcripts [57]. These up-regulated tRNAs might serve as potential novel prognostic markers in addition to their involvement in the pathogenesis of breast cancer [69].

### The modification of tRNAs in breast cancer

tRNA modifications such as tRNA base modifications and the enzymes catalyzing such modifications played an important role in the pathogenesis of breast cancer [70–72]. Studies have indicated that increased tRNA modifications enhanced the translational efficiency by modifying the anticodon swinging bases, increasing the decoding power of tRNA [33, 73]. In human breast cancer, the elevated expression of U34-modifying enzymes Elp3 and Ctu1/2, catalyzing the mcm5s2-U34 tRNA modification, directly promoted the translation of oncoprotein DEK in turn binding to the LEF1-IRES sequence to increase the translation of the oncogenic LEF-1 mRNA and promote the invasion and metastasis of breast cancer cells [72]. Meanwhile, RNA methyltransferase misu (NSUN2) had significantly increased expression level in squamous cell carcinoma, colorectal cancer and breast cancer, therefore it might act as a downstream target gene of myc and be involved in the proliferation of cancer cells [71]. Interestingly, another tRNA modification enzyme tRNA methyltransferase homolog 12 (TRMT12) was highly expressed in both several breast cancer cell lines and tissues [70], however, its elaborate molecular mechanisms were unclear.

### The mutation of tRNAs in breast cancer

Additionally, mitochondrial DNA (mtDNA) depletion and mutation had been shown to be associated with increased tumorigenic and invasive phenotype [74]. The tertiary structure of mitochondrial tRNA(mt-tRNA)was affected by its genetic mutation, which brought about severely impaired mitochondrial protein synthesis [75]. Through the analysis of clinical data of breast cancer, Meng et al. concluded that mutations in mitochondrial tRNA such as mt-tRNA^Asp^ was involved in the carcinogenesis of breast cancer [76].

### tRNA derivatives in breast cancer

Many literatures reported that tRNA derivatives were dysregulated in many malignancies including breast cancer [54, 77, 78]. Performing unsupervised analysis on normal breast epithelial cells with oncogene activation mutations and cancer cells at different stages of carcinogenesis, Veronica and his colleagues found that tsRNAs expression was modulated by oncogenes, suggesting that tsRNA might be a key effector in the pathway regulated by these oncogenes [54]. Further, tsRNA expression appeared in certain obvious stages of the process of carcinogenesis: ts-3 was strongly down-regulated in aggressive late-stage breast cancer, whereas ts-67, ts-48, and ts-6 were up-regulated only in the late-stage cancer cell line [54]. Parallelly, high abundance of tRNA-derived small RNA in breast cancer extracellular vesicles (EVs) were combined with known miR signatures of tumors to differentiate EVs from those derived from other cell sources [79]. Moreover, tRFs, which are derived from tRNA^Glu^, tRNA^Asp^, tRNA^Gly,^ and tRNA^Tyr^, compete with Y-box-binding protein 1 (YB-1) for an endogenous oncogene transcript, disrupting the stability of proto-oncogene transcripts, and reducing the expression of proto-oncogenes resulting in suppression of breast cancer progression [78]. YB-1 bound to specific miRNAs, snRNAs and tRNA-derived fragments, which might cause carcinogenic effects in breast cancer [80]. Moreover, tiRNAs cooperated with YB-1 to prevent eIF4G/A from initiating translation [21].

Clinical characteristics of breast cancer were related with changes in abundance of specific tiRNA [81]. In contrast to ER-negative tumors, ER-positive tumor showed a decline in abundance of 26 specific circulating tiRNA deriving from the isoacceptors of tRNA^Gly^, tRNA^Glu^ and tRNA^Lys^ [81]. Inflammatory breast cancer, on the other hand, was associated with increases in tiRNA-Ala in comparison to non-inflammatory breast cancer, thus suggesting that circulating tiRNA might involve in breast cancer syndromes and had potential as circulating biomarkers [81]. In ER-positive breast cancer, sex hormones and their receptors promoted the angiogenin cleavage of mature tRNA anticodon loops to produce a large number of tiRNA [77]. tiRNAs, including 5'-tiRNA^Asp^ and 5'-tiRNA^His^, had significantly higher expression in tissue or cells of breast cancer than normal epithelial counterparts [77]. Experiments manifested that specific knockdowns of 5'-tiRNA would impair cell proliferation, indicating that tiRNAs were not nonfunctionally accumulated but enhanced the cell proliferation [77]. Taken together, tRNA derivatives played different roles in different pathways.

## tRNAs and tRNA derivatives in lung cancer

### tRNAs in lung cancer

Lung cancer is one of the most life-threatening diseases with its morbidity and mortality increasing rapidly [82]. The dysregulation of tRNAs is closely related to the carcinogenesis of lung cancer. TERT, significantly enriched at tRNA^Met^, tRNA^Arg^ and tRNA^Lys^ genes, regulated expression of those tRNAs and directly controlled the rate of synthesis of global cancer proteins in various cancer cell lines, mainly including HCT116, A2780 and P493 cell line, which to some extent could promote the tumorigenesis [62].

MtDNA was more readily mutated than nuclear genomic DNA owing to the lack of protective histones, introns, and efficient DNA repair systems [83]. Indeed, mutations in the mt-tRNA gene were found to be associated with various diseases including lung cancer [84]. An increasing number of literature supported that these mt-tRNA mutations, such as tRNA^His^ A12172G, tRNA^Ala^ T5655C, tRNA^Leu^ A12330G, tRNA^Ser^ T7505C, and tRNA^Thr^ G15927A, were pathogenic and highly likely to be involved in the carcinogenesis of lung cancer [85, 86]. These mutations disrupted the secondary structure of tRNA itself, and subsequently affected tRNA post-transcriptional modifications as well as aminoacylation, which might alter the specificity or stability of the tRNA or change its affinity [87]. In all, these mutations caused a decrease in mitochondrial protein synthesis and the inability to meet the threshold of the respiratory phenotype and ATP required for normal cells [84], contributing to the tumorigenesis of lung cancer.

### tRNA derivatives in lung cancer

tRNA derivatives, including tsRNAs, tRFs and tiRNAs, are associated with lung cancer development. Pekarskya et al. found that ts-3676 and ts-4521, derived from tRNA-Thr and tRNA-Ser respectively, could act as roles of not only microRNA interacted with Argonaute proteins Ago1 and Ago2, but also P-element-induced wimpy testis (Piwi)-interacting small RNAs (piRNA) interacted with Piwi-like protein 2 (Piwil2) [44, 88]. Furthermore, using Ingenuity Pathway analysis software to evaluate changes in cancer pathways in ts-4521 cells, researchers found that the cell proliferation-related pathway and apoptosis-related pathways were associated with the absence of ts-4521 [54]. Of note, these two tsRNAs were drastically down-regulated and mutated in lung cancer samples vs. matched normal lung tissues [44]. Similarly, Balatti et al. experimentally demonstrated that overexpression of ts-46 and ts-47 significantly reduced the clonal formation in lung cancer cells, which further confirmed the involvement of those tsRNAs in lung cancer pathogenesis [54]. Additionally, the high expression of tRNA^Leu^_CAG_ derived small molecule tiRNA had a positive correlation with non-small cell lung cancer stages by promoting cell proliferation and causing G0/G1 cell cycle progression, which would be conducive to the deterioration of the cancer [89]. The down-regulation of the proto-oncogene AURKA inhibited the expression of tiRNA^Leu^ in cancer cells, suggesting that tiRNA^Leu^ might play a part in promoting the proliferation of cancer cells by regulating the expression of AURKA [89].

## tRNAs and tRNA derivatives in melanoma and other cancers

Melanoma, a type of malignant tumor derived from melanocytes common in the skin, mucous membranes, choroidal and other parts of the eye, is the most malignant skin tumor. Recently, researchers found that tumor growth and angiogenesis in 2 + tRNAi^Met^ mice was significantly faster than wild-type littermate through transfection of melanoblasts into 2 + tRNAi^Met^ transgenic mice and wild-type littermates [67]. Further study discovered that increased tRNAi^Met^ in carcinoma-associated fibroblasts drove selective and meaningful alteration of the secretion of stromal cells, especially type II collagen which provided a convenient condition for tumor growth and metastasis, whereas there was no significant effect on the cell non-secreted protein product [67]. Similarly, Birch et al. argued that the overexpression of tRNAi^Met^ in melanoma advanced cancer cell migration, invasiveness and elevated lung colonisation capacity leading to increased metastatic potential, but had a lesser impact on cell proliferation and primary tumor growth [90]. Increased tRNAi^Met^ in cancer cells relied mainly on α5β1 integrin and levels of the translation initiation ternary complex to drive cell migration and invasion. Increased tRNAi^Met^ in melanoma promoted the expression of fibronectin and α5β1integrin that were closely related to the invasion of cells [90]. Khattar et al. discovered that the increased TERT gave rise to proliferative abilities of cancer cells in melanoma, because TERT upregulated tRNA expression by its direct combination with RNA polymerase III subunit RPC32 and enhanced recruitment of chromatin resulting in an increase in the occupancy rate of RNA pol III on the tRNA gene, suggesting that TERT promoted cancer cell proliferation by augmenting tRNA expression, such as tRNA^Arg^, tRNA^Ala^, tRNA^Asn^, tRNA^Cys^, tRNA^Lys^, tRNA^Glu^ and tRNA^Thr^ [62]. In addition, there are little researches on tRNA derivatives and melanoma, which is expected to become a hot spot for future research.

The dysregulation of tRNAs and tRNA derivatives occurs in other tumors, such as cervical cancer, prostate cancer, multiple myeloma and pancreatic cancer. In cervical cancer samples, high-risk human papillomavirus (HPV) tRNA expression was significantly increased compared to other benign lesions of HPV, such as tRNA^Arg^ and tRNA^Sec^ [91]. Also, the tRFs derived from tRNA^Gln^ inhibited the translation process of the protein [92]. Dependent hormones and their receptors produced tiRNA, including 5'-tiRNA^Asp^ and 5'-tiRNA^His^ as well as 5'-tiRNA^Lys^, which also promoted the proliferation of cancer cells in prostate cancer [77]. Abnormally increased tRNAs abundance promoted translation of highly active proteins in multiple myeloma [93]. tRNA modulated MEK2 function to regulate cancer cellular behavior in pancreatic cancer [94].

## Conclusion & further perspectives

Indeed, a growing series of evidence had identified that the dysregulation of tRNAs and tRNA derivatives expression was of tremendous value and potential in cancer progression. As mentioned above, the increase of specific tRNAs and mutations of tRNAs promoted the proliferation, metastasis and invasiveness of cancer cell, as well as tumor growth and angiogenesis. An increase in tRNA-modifying enzymes would be better for tRNAs to adapt to the translation of oncogenes' preferred codons to promote cell proliferation in cancer cells. Beyond that, tRNA derivatives played an extremely important role in regulating the expression of cancer-related genes, RNA silencing and cell proliferation (Table 1).

**Table 1:** Characteristics of representative tRNAs and their derivatives in breast cancer

Cancer type	The change of the tRNA	Cell/tissue type	Function	References
ER+ breast cancer	The overexpression of tRNA^Leu^	MCF-7	Promoted cell proliferation and cell transformation	[61]
Triple-negative breast cancer	The overexpression of tRNA-Leu and tRNA-Tyr	Triple-negative breast cancer tissue	Initiated tumorigenesis	[62]
Her2(ErbB2)-positive breast	The overexpression of tRNA^Leu^_CAG_	Her2 (ErbB2)-positive breast tissue	Increased the protein synthesis and proliferative abilities of cancer	[65]
Breast cancer	The overexpression of tRNAi^Met^ and tRNA^Ile^ in immortalized human breast cancer-associated fibroblasts	MCF-7	Promoted tumor growth and angiogenesis	[67]
Breast cancer	The overexpression of tRNA^Arg^_CCG_ and tRNA^Glu^_UUC_	MCF10a, MDA-par, MDA-LM2, CN34-par, and CN-LM1a	Promoted metastasis and invasion ability	[57]
Breast cancer	Elevated mcm5s2-U34 tRNA modification	MDA-MB-231, MCF7, NMuMG	Promoted breast cancer cells invasion and metastasis	[72]
Breast cancer	The mutation of mt-tRNA^Asp^	Blood samples from breast cancer patients	Involved in the carcinogenesis of breast cancer	[76]
Breast cancer	Ts-3 was down-regulated in aggressive late-stage breast cancer, whereas ts-67, ts-48, and ts-6 were up-regulated only in the late-stage cell line	MCF7 and MDA-MB-231 cell lines	tsRNA expression appeared in certain obvious stages of the process of carcinogenesis	[54]
Breast cancer	High abundance of tRNA-derived miRNA such as miR-720 and miR-1274b	MCF7 EVs and MCF10A EVs	Served as biomarkers	[79]
Breast cancer	tRFs derived from tRNA^Glu^, tRNA^Asp^, tRNA^Gly^, and tRNA^Tyr^	MDA-MB-231, CN34 cells, CN-LM1a and MDA-LM2	Suppressed breast cancer progression	[78]
Estrogen receptor (ER)-positive breast cancer	Increased 5'-tiRNA^Asp^ and 5'-tiRNA^His^	MCF-7, BT-474	Enhanced cell proliferation	[77]
Lung cancer	TERT was significantly enriched at tRNA^Met^,tRNA^Arg^ and tRNA^Lys^ genes	A2780	TERT regulated tRNAs expression and controlled the rate of synthesis of global cancer proteins	[62]
Lung cancer	Mt-tRNA^His^, mt-tRNA^Ala^, mt-tRNA^Leu^, mt-tRNA^Ser^, and mt-tRNA^Thr^ mutation	Blood samples from lung cancer patients	Contributed to the tumorigenesis of lung cancer	[85, 86]
Lung cancer	tRNA^Thr^ and tRNA^Ser^ derived from ts-3676 and ts-4521 down-regulated and mutated in lung cancer sample	Lung cancer sample	Acted as microRNA roles and piRNA roles	[44, 88]
Lung cancer	The down-regulated of ts-46 and ts-47	A549, H1299	Significantly reduced the clonal formation of cancer cells	[54]
Lung cancer	The high expression tRNA^Leu^_CAG_ derived small molecule tiRNA	A549, H1650, PC-9, 95-D and SPCA-1 H1299 and H23	Promoted cell proliferation	[89]
Melanoma	The tRNAi^Met^ in carcinoma-associated fibroblasts	G361, BLM, LOX-IMVI	Tumor growth and angiogenesis	[67]
Melanoma	Increased tRNAi^Met^ in melanoma	Derived from the early pup skin of these Tyr: NrasQ61K/°; INK4a−/−; wild-type (wt) and Tyr: NrasQ61K/°; INK4a−/− of Melanocyte cell lines	Advanced cancer cell migration, invasiveness and lung colonisation capacity	[90]
Melanoma	TERT promoted the expression of tRNA^Arg^, tRNA^Ala^, tRNA^Asn^, tRNA^Cys^, tRNA^Lys^, tRNA^Glu^ and tRNA^Thr^	BLM, G361, LOX-IMVI	TERT promoted cancer cell proliferation by augmenting tRNA expression	[62]
Cervical cancer	tRNA^Arg^ and tRNA^Sec^ were significantly increased in HPV	Hela and W12 cell lines	The oncoproteins E6 and E7 stimulated tRNA transcription	[91]
Cervical cancer	tRF derived from tRNA^Gln^ expression was decreased in conditions of slowed cell proliferation	Hela cell	Inhibited the translation process of the protein	[92]
Androgen receptor (AR)-positive prostate cancer	Increased expression 5'-tiRNAAsp and 5'-tiRNAHis as well as 5'-tiRNALys	LNCap-FGC	Promoted the proliferation of cancer cells	[77]
Multiple myeloma	High levels of tRNA abundance such as tRNA^Arg^ and tRNA^Leu^	MM.1S, MM.1R, NCI-H929, U266 and RPMI-8266	Increased translation of highly active proteins	[93]
Pancreatic cancer	tRNA interacted with MEK2	HEK293T, Q60P, P128Q, S154F, E207K and CD18	tRNA modulated MEK2 function to regulate cellular behavior	[94]

**Fig. 2:**
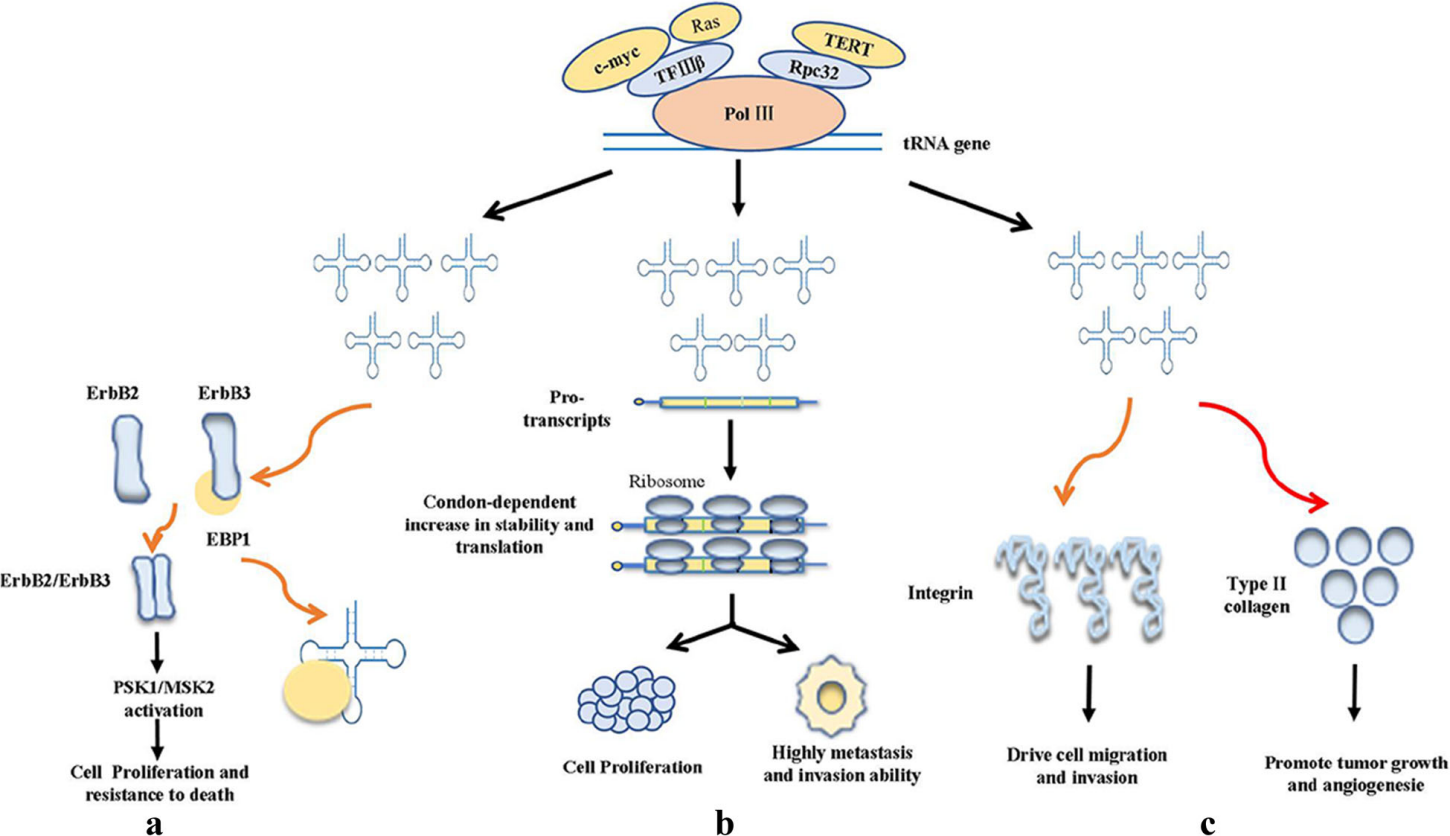
The overexpression of tRNAs regulated the progression of cancer acting on different ways: Ras, c-myc and TERT promoted the transcription of tRNA genes by binding to pol III. **a** The overexpression of tRNA activated the RSK1/MSK2 signaling pathway, thus influencing cell proliferation and cell apoptosis. **b** Overexpressed tRNA promoted the ability of invasion and metastasis by enhancing stability and translation of transcripts enriched for their cognate codons. **c** The overexpression of tRNA regulated the progression of cancer by increasing the secretion of integrin and type II collagen

tRNAs, mostly dependent on augmenting tRNA expression and acting on different pathways, regulated the progression of cancer (Fig. 2). tRNA^Leu^_CAG_ was involved in the progression of cancer by activating PSK1/MSK2 signaling pathway [65]. tRNA^Arg^_CCG_ and tRNA^Glu^_UUC_ accelerated the progress of cancer by enhancing the stability and translation of the transcripts [57]. tRNAi^Met^ was related to the development of cancer by affecting the secretion of integrin and type II collagen [67, 90]. Besides, the role of other tRNAs in cancer still require further in-depth investigation.

**Fig. 3:**
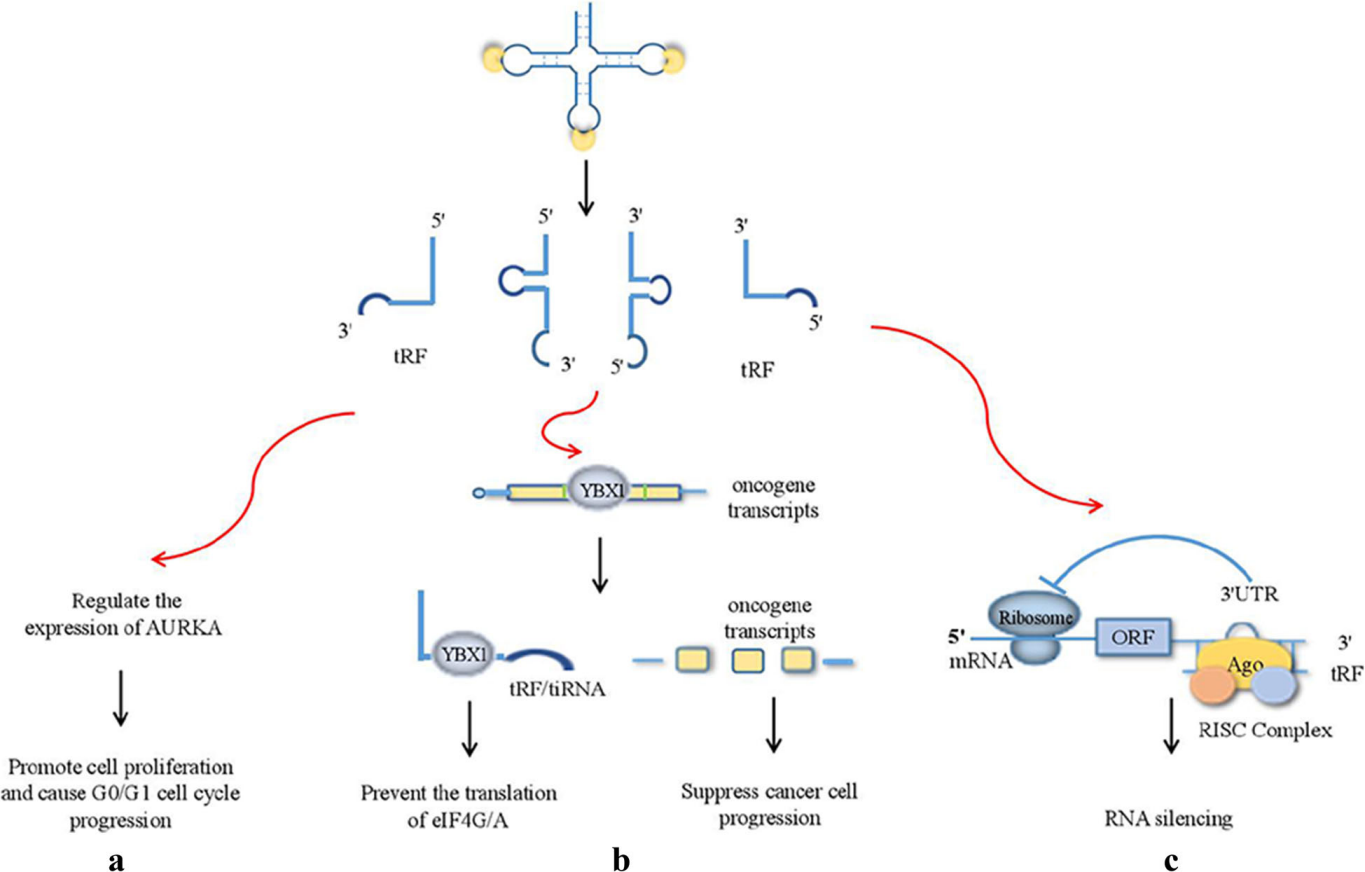
tRNA derivatives exerted their effects through different pathways. **a** tRNA derivatives promoted cell proliferation and the progression of G0/G1 cell cycle by regulating the expression of AURKA. **b** Both tRNA derivatives and endogenous oncogene transcripts competed with YBX1, suppressing the progression of cancer and preventing the translation of eIF4G/A. **c** tRNA derivatives promoted the translation of ribosomal protein mRNAs, subsequently enhancing cell proliferation and viability

Interestingly, Sun et al. recently reported that the biological functions of tRNA derivatives were significantly different in different type of cancers [95]. Andrea et al. found that 18 nt tRFs blocked reverse transcription, while 22 nt tRFs post-transcriptionally silenced coding-competent endogenous retroviruses in mouse stem cells [96]. Of note, another study by Kim et al. showed that a specific tsRNA, Leu^CAG^3'tsRNA, bound at least two ribosomal protein mRNAs (RPS28 and RPS15) to enhance their expression [97]. tRNA derivatives had double-edged sword effect on cell proliferation, whereas tRNAs mostly acted as a cell proliferation promoter (Fig. 3). Whether tRNAs may play a role in inhibiting cell proliferation needs more attention. Given that, we performed tRNAs expression profiling on normal liver tissues, adjacent tissues and liver cancer tissues, finding the different role of certain tRNAs in human hepatocellular carcinoma (HCC) tissues (data not published).

In summary, tRNA and its derivatives may serve as an effective tool for diagnosing and treating cancer. Taking tRNAs and tRNA derivatives into account will be conducive to the treatment of malignant tumors.
